# MicroRNA-27b targets CBFB to inhibit differentiation of human bone marrow mesenchymal stem cells into hypertrophic chondrocytes

**DOI:** 10.1186/s13287-020-01909-y

**Published:** 2020-09-11

**Authors:** Shuang Lv, Jinying Xu, Lin Chen, Haitao Wu, Wei Feng, Yangyang Zheng, Pengdong Li, Haiying Zhang, Lihong Zhang, Guangfan Chi, Yulin Li

**Affiliations:** 1 The Key Laboratory of Pathobiology, Ministry of Education, Department of Pathology, College of Basic Medical Sciences, Jilin University, Changchun, 130021 China; 2 Department of Gastrointestinal Surgery, Sino-Japanese Friendship Hospital of Jilin University, Changchun, 130021 China; 3 Department of Oncology, the First Hospital of Jilin University, Changchun, 130021 China; 4 Department of Bone and Joint, the First Hospital of Jilin University, Changchun, 130021 China

**Keywords:** hBMSCs, Chondrocytes, Hypertrophic differentiation, miR-27b, CBFB, RUNX2

## Abstract

**Background:**

Human bone marrow-derived mesenchymal stem cells (hBMSCs) have chondrocyte differentiation potential and are considered to be a cell source for cell-transplantation-mediated repair of cartilage defects, including those associated with osteoarthritis (OA). However, chondrocyte hypertrophic differentiation is a major obstacle for the application of hBMSCs in articular cartilage defect treatment. We have previously shown that microRNA-27b (miR-27b) inhibits hypertrophy of chondrocytes from rat knee cartilage. In this study, we investigated the role of miR-27b in chondrocyte hypertrophic differentiation of hBMSCs.

**Methods:**

Chondrogenic marker and microRNA expression in hBMSC chondrogenic pellets were evaluated using RT-qPCR and immunohistochemistry. The hBMSCs were transfected with miR-27b before inducing differentiation. Gene and protein expression levels were analyzed using RT-qPCR and western blot. Coimmunoprecipitation was used to confirm interaction between CBFB and RUNX2. Luciferase reporter assays were used to demonstrate that *CBFB* is a miR-27b target. Chondrogenic differentiation was evaluated in hBMSCs treated with shRNA targeting *CBFB*. Chondrogenic hBMSC pellets overexpressing miR-27b were implanted into cartilage lesions in model rats; therapeutic effects were assessed based on histology and immunohistochemistry.

**Results:**

The hBMSCs showed typical MSC differentiation potentials. During chondrogenic differentiation, collagen 2 and 10 (*COL2* and *COL10*), *SOX9*, and *RUNX2* expression was upregulated. Expression of miR-140, miR-143, and miR-181a increased over time, whereas miR-27b and miR-221 were downregulated. Cartilage derived from hBMSC and overexpressing miR-27b exhibited higher expression of *COL2* and *SOX9*, but lower expression of *COL10*, *RUNX2*, and *CBFB* than did the control cartilage. CBFB and RUNX2 formed a complex, and *CBFB* was identified as a novel miR-27b target. *CBFB* knockdown by shRNA during hBMSC chondrogenic differentiation led to significantly increased COL2 and SOX9 expression and decreased COL10 expression. Finally, miR-27b-overexpressing hBMSC chondrogenic pellets had better hyaline cartilage morphology and reduced expression of hypertrophic markers and tend to increase repair efficacy in vivo.

**Conclusion:**

MiR-27b plays an important role in preventing hypertrophic chondrogenesis of hBMSCs by targeting *CBFB* and is essential for maintaining a hyaline cartilage state. This study provides new insights into the mechanism of hBMSC chondrocyte differentiation and will aid in the development of strategies for treating cartilage injury based on hBMSC transplantation.

## Background

Cartilage defects, including osteochondral lesions, are common in orthopedics and are caused by trauma, necrosis, inflammation, and degeneration [1, 2]. Osteoarthritis (OA) is also one of the primary causes of cartilage defects; its global prevalence has been estimated to be over 250 million people [3]. Current therapies for cartilage defects include debridement, bone marrow stimulation, osteochondral grafting, arthroplasty, and autologous chondrocyte transplantation [4–6]. However, these treatments have limitations, such as small repair areas, limited cartilage sources, and microfractures that often cause fibrocartilage repair [7]. There is a clear need for more efficient approaches.

Accumulating evidence from basic and clinical studies demonstrates that beyond their multi-differentiation capabilities (e.g., osteogenesis, chondrogenesis, and adipogenesis), hBMSCs also have other beneficial effects, such as paracrine effects, anti-inflammatory activities, and immunomodulatory capacities. Therefore, hBMSCs have been proposed as a source for cartilage regeneration in the treatment of cartilage injury and osteochondral lesions [8, 9]. However, hBMSCs recapitulate the endochondral differentiation of growth plate chondrocytes; moreover, cells differentiated from hBMSCs tend to be hypertrophic chondrocytes that express COL10 [10] and matrix metalloproteinase 13 (MMP13) [11] and undergo mineralization, eventually leading to endochondral bone formation in vivo. This process is controlled exquisitely by cellular interactions with the surrounding matrix, growth, and differentiation factors, as well as by other environmental factors that initiate or suppress cellular signaling pathways and transcription of specific genes in a temporospatial manner [12]. During hypertrophic differentiation, multiple factors positively modulate this process from multiple signaling pathways including TGF-β, BMP, Wnt, and Indian hedgehog (IHH), as well as transcription factors including Runx2 and MEF2C [13, 14]. Additionally, noncoding RNAs including microRNAs (miRNAs) and long noncoding RNAs (LncRNAs) participate in and control this processing [15, 16]. However, the underlying mechanisms by which they influence differentiation have not been fully elucidated. Hence, it is necessary to study the mechanisms underlying hypertrophic chondrocyte differentiation to help design strategies to inhibit this process and maintain the normal chondrocyte phenotype.

MicroRNAs (miRNAs) are a class of noncoding small endogenous RNAs (20–24 nucleotides in length) that either suppress translation or degrade target mRNAs through binding to their 3′-untranslated regions (UTRs) [17]. They play critical roles in multiple cellular processes including differentiation, development, proliferation, and tumorigenesis [18]. Generally, a single miRNA regulates multiple target genes and can have profound effects on key signaling pathways. They are also involved in chondrogenic differentiation of hBMSCs. For example, miR-29b, possibly via its target gene histone deacetylase 4 (*HDAC4*), promotes the hypertrophic phenotype [19]. Overexpression of miR-145 in C3H10T1/2 cells reduced SOX9 expression, thus significantly suppressing cartilage matrix synthesis in vitro [20]. Additionally, miR-495, miR-92a, miR-483, miR-892b, and miR-218 have been found to play important roles in hBMSC chondrogenic differentiation [21–25]. Whether other miRNAs are involved in this process has not yet been clearly elucidated.

In a previous study, we found that in chondrocytes from knee cartilage of 1–3-day-old rat pups, miR-27b inhibited hypertrophy by targeting peroxisome proliferator-activated receptor γ2 (PPARγ2) [26]. MMP13, which is a hypertrophic chondrocyte marker, is a direct target of miR-27b [27]. In a preliminary study, following induction of chondrogenic differentiation in rabbit BMSCs through high-density pellet culture for 21 days, we observed that miR-27b expression in the BMC-derived chondrocytes was significantly lower than that in normal chondrocytes isolated from knee cartilage of the same donor. Therefore, we hypothesized that miR-27b may play a role in hypertrophic differentiation of hBMSC-derived chondrocytes.

Core-binding factor, β-subunit (CBFB) is a non-DNA-binding partner of the Runt-related transcription factors1–3 (RUNX1–3). It cooperates with these factors to form DNA-protein complexes and protects them from degradation [28, 29]. CBFB is involved in skeletal development and osteoblast and chondrocyte differentiation, as well as in fracture healing [30, 31]. RUNX2 is an essential transcription factor involved in osteogenesis and chondrocyte hypertrophy. In articular cartilage, RUNX2 is extensively expressed in prehypertrophic and hypertrophic chondrocytes. RUNX2 regulates expression of COL10, SPP1, IBSP, MMP13, and VEGFA, all critical markers for hypertrophic chondrocytes [32, 33]. Thus, RUNX2 most likely functions during hBMSC chondrogenic differentiation. Therefore, it would be interesting to investigate whether downregulation of RUNX2 or/and CBFB would block or delay hypertrophic differentiation. Using the target prediction program TargetScan, we found that the 3′-UTR of *CBFB* mRNA, but not that of *RUNX2* mRNA, has putative miR-27b-binding sites. Based on this finding, we hypothesized that *CBFB* may be a direct target of miR-27b and that by modulating *CBFB* expression, miR-27b may inhibit hypertrophy during hBMSC chondrogenic differentiation.

In this study, we aimed to determine the expression pattern and function of miR-27b during hBMSC chondrogenic differentiation, which are crucial for overcoming the obstacle of hypertrophic differentiation and will aid in improving the therapeutic efficacy of hBMSC-derived chondrocytes in the repair of hyaline cartilage defects or OA.

## Methods

### Human subjects

1Human bone marrow aspirates were obtained from patients undergoing elective bone surgery (*n* = 5 donors, age 30–60 years, 3 men, 2 women). All participants were informed regarding the nature of this study and gave written informed consent. The study was approved by the Ethics Committee of The First Hospital of Jilin University (approval 2017-342).

### Isolation and culture of hBMSCs

Twenty-milliliter aliquots of bone marrow suspension were centrifugated and washed twice with PBS. Cells were resuspended in DMEM/F12 (1:1, HyClone, USA) containing 10% fetal bovine serum (FBS; Ausbian, Australia) and 10 ng/ml basic fibroblast growth factor (bFGF; Active Bioscience, Germany) and seeded onto culture dishes. Cultures were maintained in an incubator with 5% CO_2_ at 37 °C. Once cells reached > 90% confluence (passage 0), they were passaged using 0.25% trypsin/ethylenediaminetetraacetic acid (EDTA). Cells from passage 4 were used in the experiments. Samples from the five individuals were processed independently.

### Flow cytometry

In brief, hBMSCs were trypsinized and washed with phosphate-buffered saline (PBS) twice. The cell concentration was adjusted to 0.5 × 10^5^ per tube. Cells were incubated with FITC-labeled antibodies against CD34, CD44, CD45, CD73, CD90, and CD105 (Bioscience, USA) at 4 °C in the dark for 30 min. Cells were washed with PBS twice and centrifuged. The cells were fixed with 4% paraformaldehyde and analyzed in a FACS–Canto™ II Flow Cytometer (BD Biosciences).

### Chondrogenic differentiation induction

Cells (2.5 × 10^5^) were resuspended in 0.5 ml of cartilage-inducing medium, high-glucose DMEM (HyClone), 100 nM dexamethasone, 100 μg/ml sodium pyruvate, 25 mg/ml vitamin C, 40 mg/ml d-valine, 10 ng/ml transforming growth factor β3 (TGF-β3), and 1% ITS (Sigma-Aldrich), and pelleted. The pellet was incubated in an incubator with 5% CO_2_ at 37 °C. The medium was refreshed every 2 days and chondrogenic pellets were harvested after 7, 14, and 21 days. The harvested pellets were fixed with 4% paraformaldehyde for 72 h, embedded in paraffin, sectioned at 5-μm thickness, and stained with Alcian blue (Cyagen, China) and Safranin O (Sigma-Aldrich). Tissue organization and extracellular matrix deposition were analyzed by microscopy (Olympus, Japan).

### Vector construction and packaging

The pLV3 shuttle (Jima, China) and packaging (pGag/Pol, pRev, and pVSV-G) plasmids were transfected into 293T cells (Cell Bank of the Chinese Academy of Sciences, China) using RNAi-Mate (Jima, China). The sequences harbored in the pLV3 shuttle plasmid were as follows: miR-27b (full-length 5′-ACCUCUCUAACAAGGUGCAGAGCUUAGCUGAUUGGUGAACAGUGAUUGGUUUCCGCUUUGUUCACAGUGGCUAAGUUCUGCACCYGAAGAGAAGGUG-3′); shRNA-*CBFB* (GGAGGCAAGAAGACAACAAGA). Cells were cultured in DMEM/F12 (HyClone) medium with 10% FBS (Ausbian) for 48 h. The lentivirus-containing medium was collected, and the virus was concentrated using Centricon centrifugal filters (Beckman, USA).

### Lentiviral transduction and selection

For lentiviral transduction, hBMSCs prior to differentiation were seeded in 6-well culture plates at 2 × 10^5^ cells/well and cultured in 10% FBS (Ausbian)-containing DMEM/F12 (HyClone) for 24 h. Then, the cells were transfected with lentiviral at a multiplicity of infection of 50 in medium containing 5 μg/ml polybrene for 12 h, after which the medium was refreshed. Puromycin (1 μg/ml) was added to the medium for 3 days to select stably transfected cells.

### Mimic-mediated overexpression of miR-27b and siRNA-mediated knockdown of β-catenin in hBMSCs

Cells were seeded in 6-well plates at 2 × 10^5^/well and cultured for 24 h. Pre-miR-27b mimic (Ambion, USA) was delivered into the cells using DharmaFECT1 (Life Technologies, Germany) following the manufacturer’s protocol. Briefly, mimics (100 nM) were mixed with the transfection agent in Opti-MEM (Invitrogen, USA), incubated for 20 min, and added to serum-free medium. As controls, cells were transfected with pre-miRNA precursor mimics (Ambion, USA) or treated with transfection reagents alone. Twenty-four hours later, the medium was replaced with cartilage-inducing medium. Cells were cultured for another 48 h for RT-qPCR and western blotting. The transfection procedure of siRNA is in line with that of mimics.

### RT-qPCR

RNA was extracted from cells using TRIzol (Invitrogen, USA). Expression levels of miRNAs were measured with the All-in-One miRNA RT-qPCR Detection Kit (GeneCopoeia, USA). *U6* was used as a reference gene for normalization. Samples were analyzed on an ABI 7300 qPCR instrument (Applied Biosystems, USA). RT-qPCR reactions for *COL2*, *COL10*, *CBFB*, *MMP13*, *PPARγ*, and *SOX9* were run using TransStart Green qPCR SuperMix (TransGen, China) in the ABI7300 qPCR instrument. PCR primer sequences are listed in Table 1. Gene expression was normalized to that of *GAPDH* and quantified using the 2^–ΔΔCt^ method.

**Table 1:** Primers of qPCR

Gene	Forward primers (5′ to 3′)	Reverse primers (3′ to 5′)
CBFB	GGCGCGGCCTGAGGGCGGGAAGA	CGTTAAGTGGAGCACAGCTTATG
COL2	CGTCCAGATGACCTTCCTACG	TGAGCAGGGCCTTCTTGAG
COL10	GGCAGAGGAAGCTTCAGAAA	AAGGGTATTTGTGGCAGCATA
GAPDH	CCATGTTCGTCATGGGTGTGA	CATGGACTGTGGTCATGAGT
MMP13	GAGTTGGACTCACTGTTGGTC	GCAAGAGTCACAGGATGGTAG
PPARγ	CTGGCCTCCCTGATGAATAA	CGCAGGTTTTTGAGGAACTC
RUNX2	TTACTGTCATGGCGGGTAAC	AGGTGAAACTCTTGCCTCGT
SOX9	GTACCCGCACTTGCACAAC	TCTCGCTCTCGTTCAGAAGTC
hsa-mir-27b	HmiRQP0361GeneCopoeia, China	
hsa-mir-140	HmiRQP0182GeneCopoeia, China	
hsa-mir-143	HmiRQP0188GeneCopoeia, China	
hsa-mir-145	HmiRQP0191GeneCopoeia, China	
hsa-mir-181a	HmiRQP0231GeneCopoeia, China	
hsa-mir-221	HmiRQP0338GeneCopoeia, China	

### Western blotting

Cells were harvested and total proteins were extracted using RIPA buffer (Beyotime Biotechnology, China). Protein concentrations were assessed using a Pierce BCA Assay kit (Beyotime Biotechnology). Total protein (30 μg) was loaded onto 10% sodium dodecyl sulfate polyacrylamide gels, electrophoresed, and transferred to a polyvinylidene fluoride membrane. The membrane was blotted with antibodies against COL2 (1:1000, Abcam, USA), SOX9 (1:2000, Sigma-Aldrich), RUNX2 (1:500, Sigma-Aldrich), MMP13 (1:1000, Abcam), CBFB (1:1000, Santa Cruz Biotechnology, USA), COL10 (1:2000, Abcam), β-catenin (1:1000, CST, USA), PPARγ (1:1000, CST, USA), and GADPH (1:2000, TransGen) at 4 °C overnight, followed by incubation with peroxidase-conjugated secondary anti-mouse or anti-rabbit IgG antibody (1:2000, TransGen) at room temperature for 60 min. Signals were detected by chemiluminescence using the ECL-Plus detection system (TransGen). Protein bands were semi-quantified based on three independent experiments by densitometry using Bandscan 5.0 (Glyko Biomedical, USA) software. GAPDH was used as a loading control.

### Luciferase reporter assay

The 3′-UTR of *CBFB* and point mutations were PCR-amplified with primers flanked by *Xho*I and *Not*I restriction sites. The fragment was cloned into the pSICHECK2 vector (Promega, USA) downstream of the Renilla luciferase gene in sense and antisense orientations. Chondrocytes were seeded into 96-well plates and co-transfected with the luciferase reporter constructs and 75 nM pre-miR-27b precursor, using DharmaFECT1. Luminescence was measured 48 h post transfection using the Dual-Glo Luciferase Assay System (Promega).

### Coimmunoprecipitation (co-IP)

Chondrogenic differentiation was induced as described above. Cells were washed with PBS, harvested from the culture dishes, and centrifuged. Cells were lysed in ice-cold IP Lysis/Wash Buffer (TransGen) containing protease inhibitors. Part of the lysate was used as an input control for precipitation, while the remainder was washed with TBST (Tris 10 mM, NaCl 150 mM, Tween-20 1%, pH 7.5) three times and used for co-IP with 2 μg anti-CBFB antibody (1:1000, CST) or anti-RUNX2 antibody (1:500, CST). After 3 h of incubation, Protein G Sepharose was added and the samples were incubated overnight at 4 °C, then centrifuged at 12,000×*g* for 1 min. The precipitates were rinsed with IP buffer (0.5% NP-40, Tris 20 mM, pH 8.0, 150 mM NaCl) four times to remove non-specific binding molecules. IgG was used as a NC for precipitation. Relative expression levels of CBFB and RUNX2 in the precipitates were assessed by western blot.

### Articular cartilage defect rat model and cartilage pellet transplantation

Animal surgeries were performed according to protocols approved by the Jilin University Institutional Animal Care and Use Committee and following guidelines for the care and use of laboratory animals (approval 2019064). Eighteen specific pathogen-free male Sprague-Dawley rats (2–3 months of age, 200–250 g) were randomly allocated into three groups: a NC group, a miR-27b-SC-LV (miR-27b-scramble-LV) group, and a miR-27b-LV group (*n* = 6 in each group). NC group cells were treated with transfection reagents alone. Transfected hBMSCs were induced to differentiate in culture for 14 days. Then, chondrogenic pellets were harvested for transplantation in vivo.

The right hind limb of each animal was used for defect preparation and treatment. Rats were anesthetized by intraperitoneal injection of 40 mg/kg sodium pentobarbital. After shaving and disinfection, the right knee joint was exposed through a medial parapatellar approach. The patella was dislocated laterally, without patellar eversion. A defect (1.5-mm diameter and 1.5-mm depth) was created in the trochlear groove of the femur cartilage using a dental drill. Debris was removed from the defect using a curette and irrigation. Then, a chondrogenic pellet was implanted in the lesion, the patella was physically relocated, and the joint capsule, subcutaneous tissue, and skin were closed with sutures. All rats were returned to their cages after surgery and allowed to move freely. During the experiment, cyclosporine (Novartis, Switzerland) at a dose of 10 mg/kg was intraperitoneally administered on days − 2, − 1, 0, 1, 4, 7, and 10 [34]. At 4 weeks post transplantation, rats were sacrificed and their femurs excised and fixed using 10% formalin for histological examinations.

### Histochemistry and immunohistochemistry

Femoral samples were fixed in 10% buffered formalin, decalcified in 10% buffered EDTA (pH 7.4, Sigma-Aldrich), embedded in paraffin, and sectioned at 5-μm thickness. Hematoxylin and eosin (HE) staining was employed to evaluate the histological structure, and both toluidine blue and Safranin O were utilized to visualize proteoglycans and glycosaminoglycan (GAG) deposition [35]. For immunohistochemistry (IHC), after deparaffinization, rehydration, and several washes with PBS, sections were stained using the S-100 Protein Detection Kit (Fuzhou Maixin Biotech, China). Then, the sections were incubated with antibodies against COL1 (1:200, Abcam), COL2 (1:100, Abcam), SOX9 (1:200, Sigma-Aldrich), COL10 (1:200, Abcam), anti-human-nuclei antibody (1:1000, CST), osteopontin (OPN) (1:100, Abcam), and osteocalcin (OCN) (1:100, Bio-Techne, China) stained with 3,3-diaminobenzindine and counterstained with hematoxylin. The integrated optical density was semi-quantitated using ImageJ 1.52 software (Media Cybernetics, USA). The stained histological samples were imaged and analyzed to determine the extent of cartilage repair. Cartilage repair was graded blindly by two observers using the O’Driscoll score [36].

### Statistical analysis

All data are presented as means ± standard deviations from at least three independent experiments. Statistical analyses were performed using GraphPad Prism 7.0 software. Means of two groups were compared using independent-samples *t*-tests, and means of multiple groups were compared by one-way analyses of variance. For histological scoring of cartilage repair in vivo, the nonparametric Mann-Whitney *U* test was used to establish the statistical significance of the graded samples across independent observations of the repaired tissue made by two different observers. *P* < 0.05 was considered statistically significant.

## Results

### Cultured hBMSCs expressing typical MSC markers

**Fig. 1:**
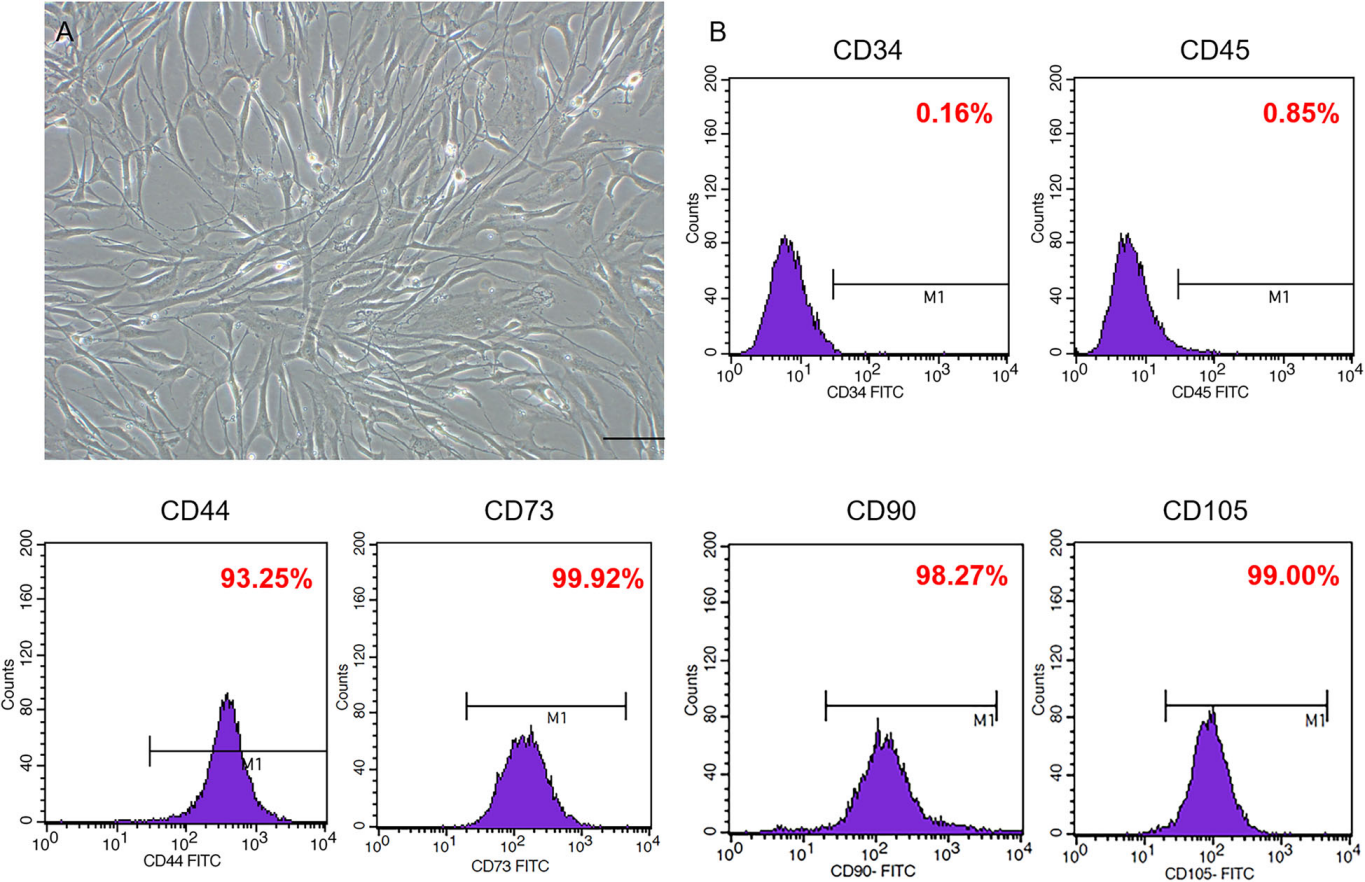
Identification of morphology and surface markers of hBMSCs. **a** Morphology, resembling typical fibroblast-like cells. Scale bar = 100 μm. **b** Flow-cytometric analysis of hBMSC surface markers

First, we evaluated whether the cultured cells were hBMSCs. The cultured cells showed the typical spindle-shaped morphology of hBMSCs (Fig. 1a). Flow cytometry indicated that the cells were positive for CD44 (93.25%), CD73 (99.92%), CD90 (98.27%), and CD105 (99%), but negative for CD34 (0.16%) and CD45 (0.85%) (Fig. 1b). These results showed that the isolated and cultured cells exhibited typical MSC morphology and markers expression.

### Hypertrophic chondrocyte differentiation of cultured hBMSCs occurs after 2 weeks of induction

Hypertrophic changes reportedly occur after chondrogenic induction of hBMSCs for 2 weeks [37]. We induced chondrogenic differentiation by the pellet culture method for 3 weeks and evaluated the expression of chondrogenic and hypertrophic markers at 0, 7, 14, and 21 days to determine the timing of hypertrophy.

**Fig. 2:**
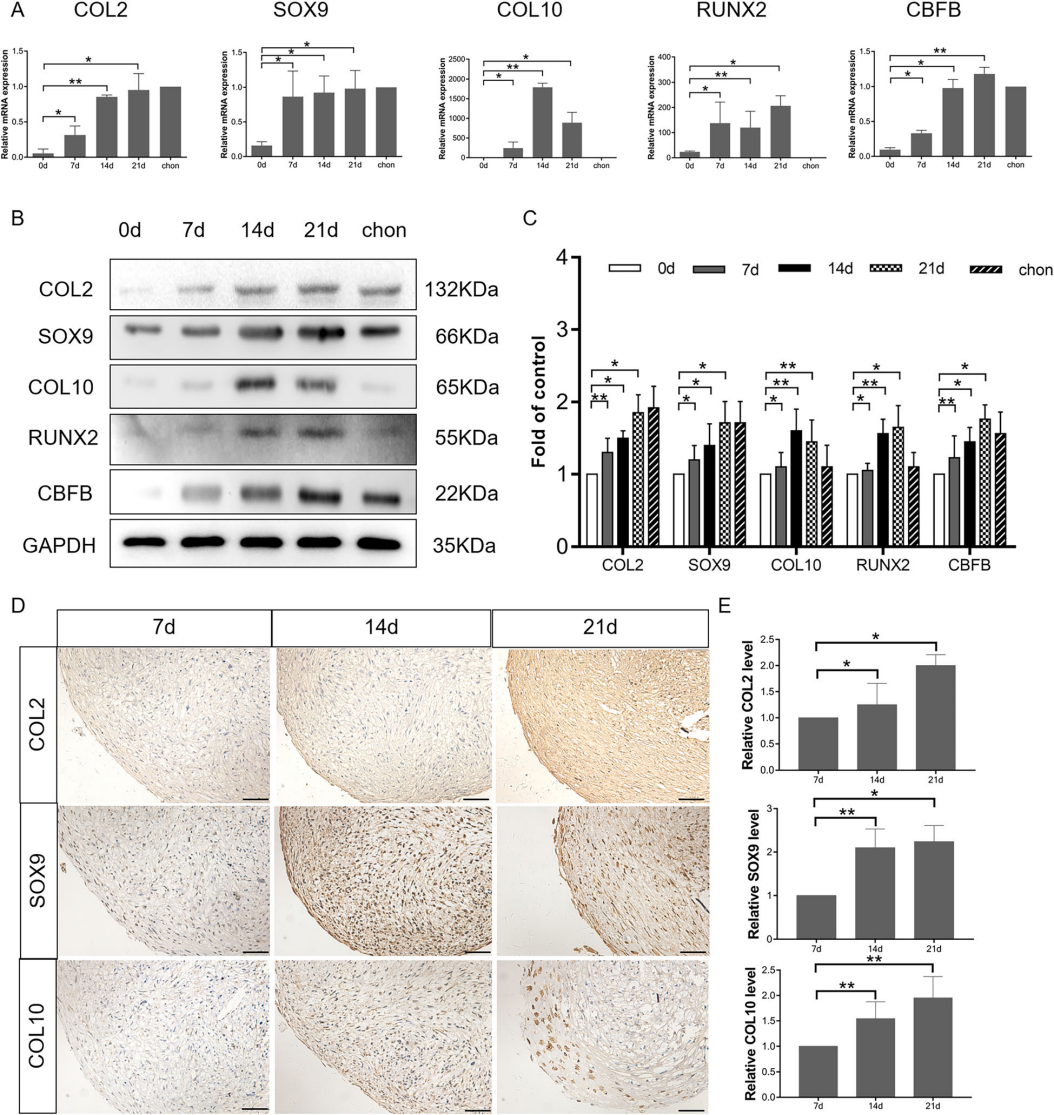
Confirmation of chondrogenic differentiation of hBMSC chondrogenic pellets. **a** mRNA expression of *COL2*, *SOX9*, *COL10*, *RUNX2*, and *CBFB* at days 0, 7, 14, and 21, as well as in control chondrocytes (chon) as measured by RT-qPCR. *P < 0.05, **P < 0.001. **b** Western blot analysis of expression of COL2, SOX9, COL10, RUNX2, and CBFB in hBMSC-derived cartilage pellets on 0, 7, 14, and 21 days. **c** Semi-quantification of western blot data. *P < 0.05, **P < 0.001. **d** IHC for COL2, SOX9, and COL10 in cartilage pellets on 7, 14, and 21 days. Scale bar = 50 μm. **e** Optical density for COL2, SOX9, and COL10 was evaluated by ImageJ software, and the data represented the expression levels of COL2, SOX9, and COL10. *P < 0.05, **P < 0.001

We used RT-qPCR to detect the mRNA levels of *COL2*, *SOX9*, *COL10*, *RUNX2*, and *CBFB* in cartilage pellets at 0, 7, 14, and 21 days. *COL2* (*P* < 0.05) and *SOX9* (*P* < 0.05) (hyaline cartilage markers) mRNA levels significantly increased on day 7 over those on day 0. In addition, the mRNA expression of the hypertrophy markers *COL10* (*P* < 0.001) and *RUNX2* (*P* < 0.001) significantly increased on day 14 (Fig. 2a). Western blot analysis of the hBMSC-derived cartilage pellets revealed that the expression of COL2 (*P* < 0.001) and SOX9 (*P* < 0.05) was markedly increased on day 7, and the levels of COL10 (*P* < 0.001) and RUNX2 (*P* < 0.001) were significantly increased on day 14 (Fig. 2b, c). In addition, from IHC analysis, we found that in comparison with pellets on day 7, after 14-day and 21-day induction, COL2 (P < 0.05), SOX9 (P < 0.001), and COL10 (P < 0.001) expression increased respectively (Fig. 2d, e), suggesting that hBMSCs do tend to acquire a hypertrophic phenotype after chondrogenic induction in culture from day 14.

### miR-27b expression is downregulated during hypertrophic chondrocyte differentiation

**Fig. 3:**
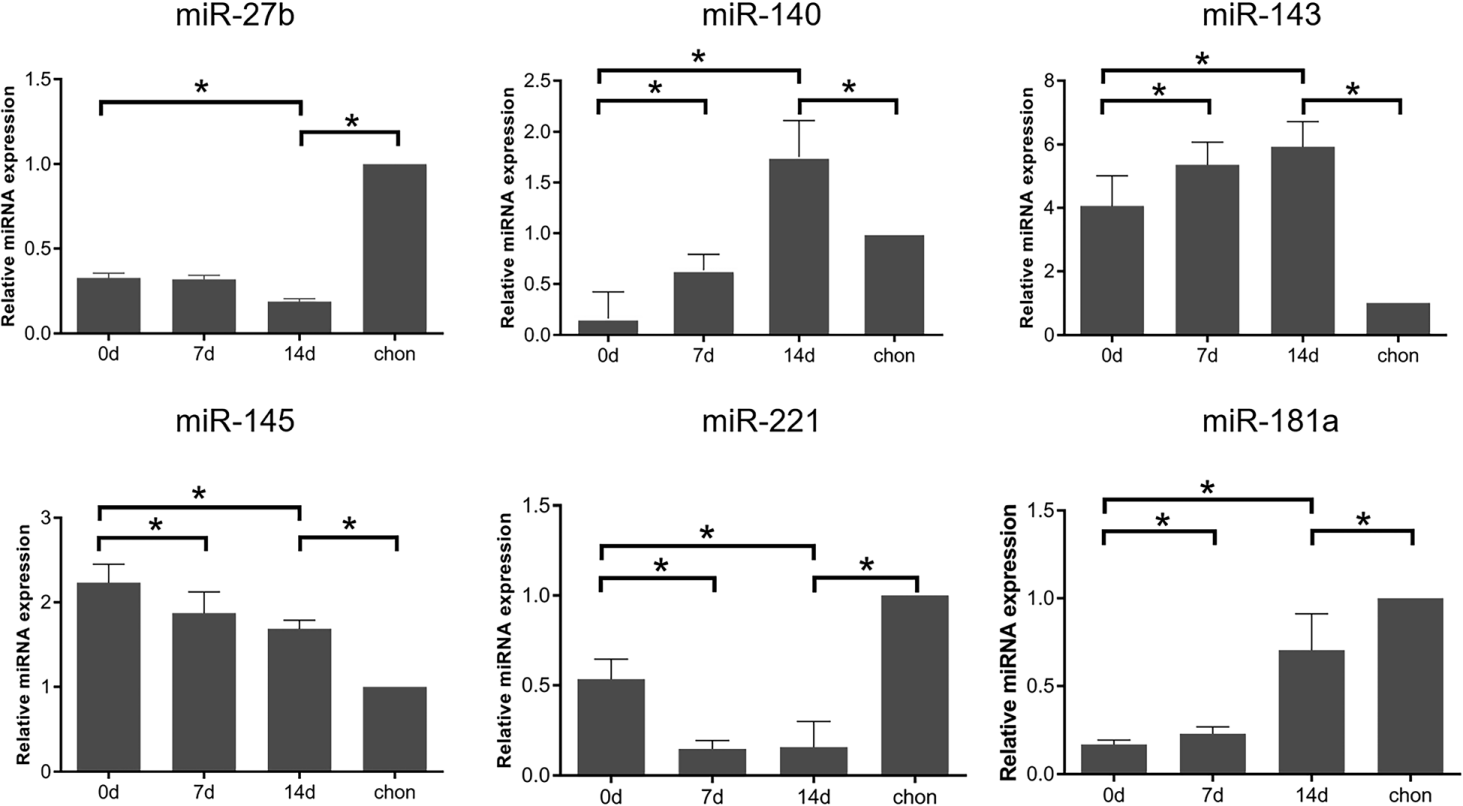
miRNA expression during hypertrophic chondrocyte differentiation. Changes in miRNA expression were assessed by RT-qPCR. Chondrocyte pellets were harvested on days 0, 7, and 14. Data are shown as fold changes. *P < 0.05

Certain miRNAs (e.g., miR-140, miR-143, and miR-145) have dramatically altered expression and play critical roles during chondrogenic differentiation of hBMSCs [20, 38–40]. Therefore, we evaluated their expression during chondrogenic differentiation. The expression of miR-140 (*P* < 0.05), miR-143 (*P* < 0.05), and miR-181a (*P* < 0.05) gradually increased on days 7 and 14 relative to their levels on day 0. The expression of miR-140 (*P* < 0.05) and miR-143 (*P* < 0.05) was significantly higher in hBMSC-derived cartilage pellets than in normal chondrocytes. In contrast, miR-181a (*P* < 0.05) was expressed at a lower level in cartilage pellets than in normal chondrocytes. The miR-145 (*P* < 0.05) level tended to slightly decrease over time, but was higher in cartilage pellets than in normal chondrocytes. Both miR-27b (*P* < 0.05) and miR-221 (*P* < 0.05) had significantly decreased expression on day 14, but were consistently expressed at lower levels in cartilage pellets than in normal chondrocytes (Fig. 3). These results suggested that miR-143, miR-145, and miR-181a may be involved in hBMSC hypertrophic chondrocyte differentiation, whereas miR-27b and miR-221 may negatively regulate this process.

### Overexpression of miR-27b inhibits hypertrophic chondrocyte differentiation of hBMSCs

**Fig. 4:**
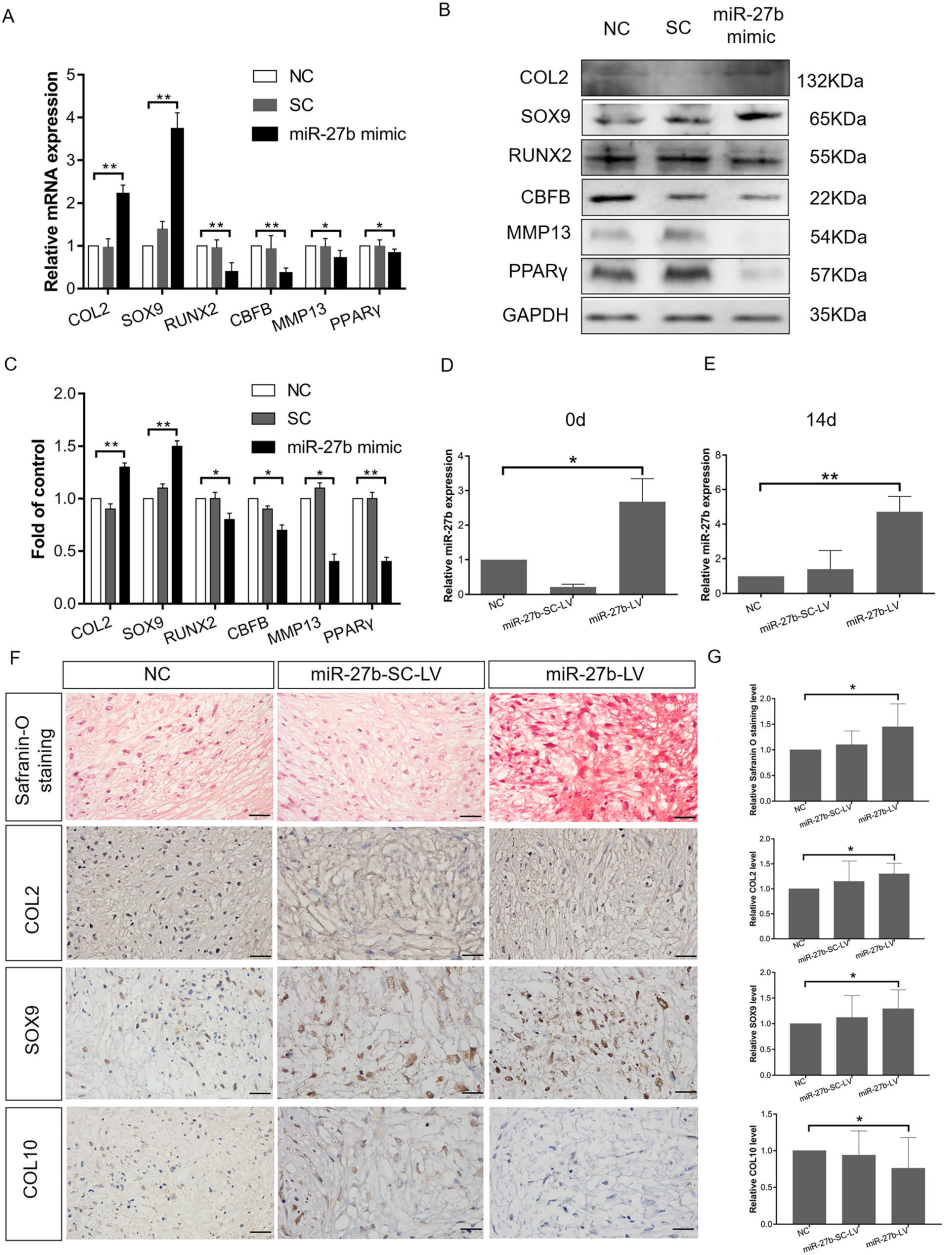
miR-27b overexpression inhibits hypertrophic differentiation of hBMSC-derived chondrocytes. **a** Expression of COL2, SOX9, RUNX2, CBFB, MMP13, and PPARγ upon overexpression of miR-27b mimic in hBMSCs as measured by RT-qPCR on day 3. *P < 0.05, **P < 0.001. **b** Western blot measurement of expression of COL2, SOX9, RUNX2, CBFB, MMP13, and PPARγ expression in miR-27b-overexpressing hBMSCs compared to NC on day 3 after transfection. **c** Semi-quantification of western blot data. *P < 0.05, **P < 0.001. **d** miR-27b expression on day 0 after miR-27b-LV and miR-27b-SC-LV transfection followed by chondrogenic induction, as measured by RT-qPCR. *P < 0.05. **e** miR-27b expression on day 14 after miR-27b-LV and miR-27b-SC-LV transfection followed by chondrogenic induction for 14 days, as measured by RT-qPCR. **P < 0.001. **f** Safranin O staining and IHC for COL2, SOX9, and COL10 in cartilage pellets on day 14. Scale bar = 20 μm. **g** Safranin O staining was performed to measure GAGs. Optical density for GAGs was evaluated by ImageJ software, and the data represented GAG content. Optical density for COL2, SOX9, and COL10 was evaluated by ImageJ software, and the data represented the expression levels of COL2, SOX9, and COL10. *P < 0.05

We previously found that miR-27b expression was inversely correlated with chondrocyte hypertrophic differentiation in postnatal rat knee articular cartilage [26]. Therefore, we next focused on its during hBMSC chondrogenic differentiation in culture*.* The hBMSCs were incubated with a chemically synthesized miR-27b mimic for 3 days to exogenously enhance miR-27b expression. COL2 (*P* < 0.001) and SOX9 (*P* < 0.001) mRNA levels were significantly upregulated, whereas the mRNA expression levels of RUNX2 (*P* < 0.001), CBFB (*P* < 0.001), MMP13 (*P* < 0.05), and PPARγ (*P* < 0.05) were significantly downregulated (Fig. 4a). In accordance, western blot analysis of the hBMSC-derived cartilage pellets revealed that the protein expression of COL2 (*P* < 0.001) and SOX9 (*P* < 0.001) was markedly increased in the miR-27b-overexpressing hBMSCs relative to NC, whereas the levels of RUNX2 (*P* < 0.05), MMP13 (*P* < 0.05), PPARγ (*P* < 0.001), and CBFB (*P* < 0.05) were significantly decreased (Fig. 4b, c). This finding supported our hypothesis that miR-27b may be promoting chondrogenesis and inhibiting hypertrophy.

We then utilized a miR-27b-LV to enhance miR-27b expression in hBMSCs and induced chondrogenic differentiation by pellet culture. RT-qPCR confirmed that after transfection, miR-27b expression was upregulated 4.7-fold (*P* < 0.001) and was maintained at a high level for up to 14 days (Fig. 4d, e). Cartilage pellets after 14-day induction were fixed and subjected to IHC for COL2, SOX9, and COL10 (Fig. 4f, g). We found that overexpression of miR-27b promoted the expression of COL2 (*P* < 0.05) and SOX9 (P < 0.05) when compared with the levels in cells treated with miR-27b-LV, miR-27b-SC-LV, or NC. In contrast, COL10 (*P* < 0.05) expression declined slightly in the miR-27b-overexpression group relative to that in the NC groups. These results suggested that overexpression of miR-27b in hBMSCs may effectively inhibit hypertrophic differentiation after chondrogenic induction in culture.

### miR-27b inhibits hBMSC hypertrophic chondrocyte differentiation by regulating CBFB and possibly upregulates SOX9 expression by stimulating β-catenin expression

**Fig. 5:**
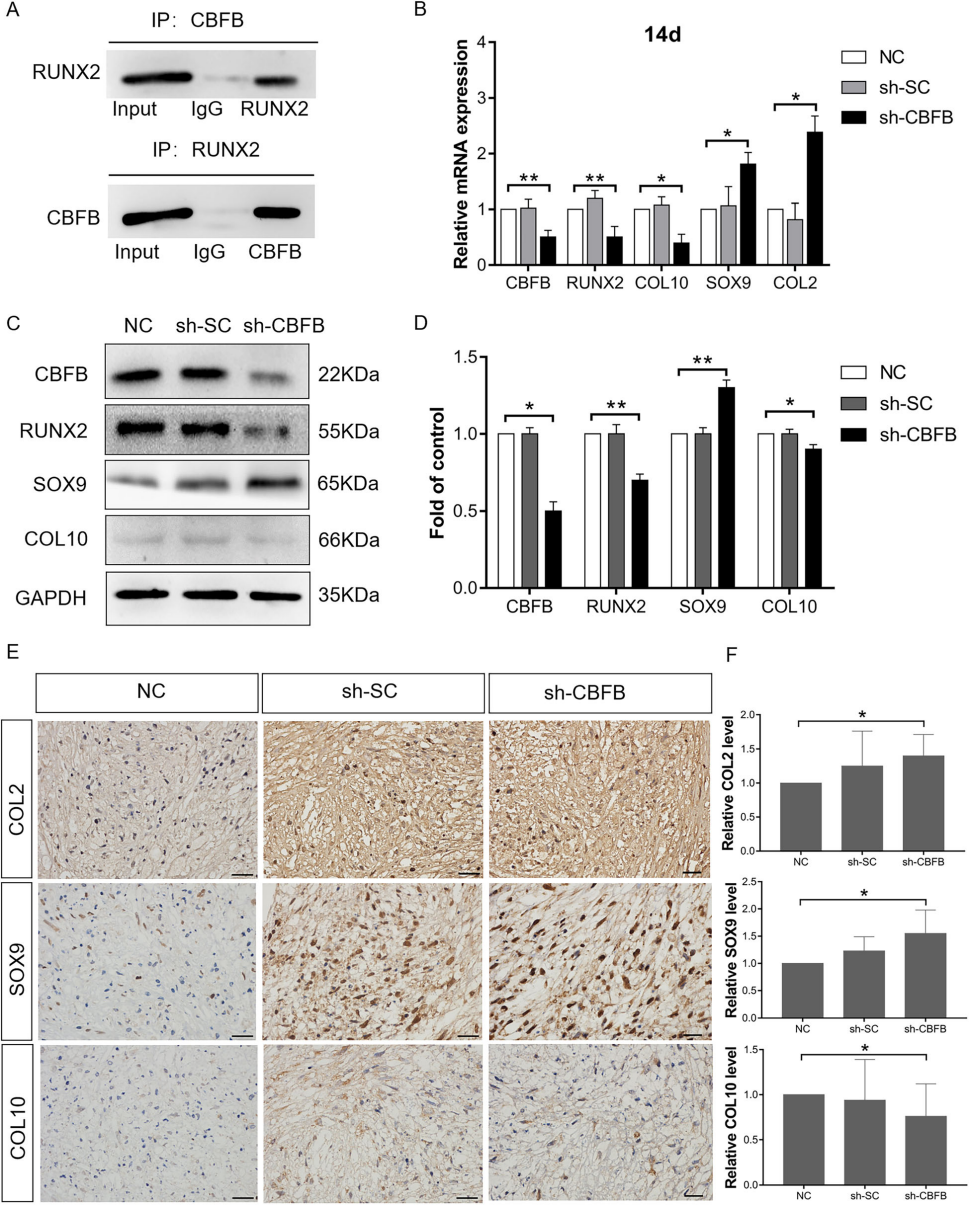
miR-27b inhibits hypertrophic differentiation of hBMSC-derived chondrocytes and upregulates SOX9 expression. **a** Protein levels of CBFB and RUNX2 in coimmunoprecipitates. **b** After sh-CBFB and sh-SC transfection, on 14 days mRNA expression of *CBFB*, *RUNX2*, *COL10*, *SOX9*, and *COL2* as measured by RT-qPCR. *P < 0.05, **P < 0.001. **c** Protein levels of CBFB, RUNX2, SOX9, and COL10 as measured by western blot. **d** Semi-quantification of western blot data. *P < 0.05, **P < 0.001. **e** IHC staining of COL2, SOX9, and COL10 expression in chondrogenic pellets transfected with NC, sh-SC, and sh-CBFB. Scale bar = 20 μm. **f** Measurement of optical density for COL 2, SOX9, and COL10 was evaluated by ImageJ software, and the data represented the expression levels of COL 2, SOX9, and COL10. *P < 0.05

RUNX2 is an essential transcription factor for skeletal development that plays an important role in chondrocyte hypertrophy [14]. CBFB is a co-transcription factor of RUNX2; they heterodimerize to enhance DNA binding and stability of RUNX2 and inhibit its ubiquitination [41]. Co-IP was used to verify that during chondrogenic differentiation of hBMSCs, CBFB directly interacts with RUNX2 to form a complex. When total lysates of hBMSC cartilage pellets were immunoprecipitated with CBFB antibody, RUNX2 expression was detected by western blot. Conversely, when the same total lysates were immunoprecipitated with RUNX2 antibody, CBFB was present (Fig. 5a). These results demonstrated that during hBMSC chondrogenic differentiation, RUNX2 functions may rely on heterodimerization with CBFB.

We then utilized shRNA to silence *CBFB* (Fig. 5b, c). Fourteen days after shRNA transfection, *COL10* (*P* < 0.05) and *RUNX2* (*P* < 0.001) mRNA expression was significantly downregulated, whereas *SOX9* (*P* < 0.05) and *COL2* (*P* < 0.05) mRNA levels were significantly upregulated. Consistent with the mRNA data, western blotting showed that after shRNA treatment, the protein levels of RUNX2 (*P* < 0.001) and COL10 (*P* < 0.05) were significantly downregulated, with SOX9 (*P* < 0.001) protein levels significantly increased relative to the levels in NC pellets (Fig. 5c, d). Furthermore, IHC revealed that, in line with the western blot results, *CBFB*-silenced pellets had stronger expression of COL2 (*P* < 0.05) and SOX9 (*P* < 0.05) and weaker expression of COL10 (*P* < 0.05) than NC pellets (Fig. 5e, f). These results showed that blocking CBFB expression effectively inhibited hypertrophic chondrocyte differentiation of hBMSCs.

SOX9 is a negative regulator of chondrocyte hypertrophic differentiation [42], and the β-catenin pathway is involved in controlling SOX9 expression [43]. Thus, we examined whether miR-27b enhanced SOX9 expression through the β-catenin pathway. After miR-27b mimic treatment, SOX9 (*P* < 0.001) and β-catenin (*P* < 0.05) protein levels were both upregulated (Additional file 2: Fig. S2A and B). During chondrogenic differentiation, we treated all groups of hBMSCs with miR-27b mimics and simultaneously transfected with siRNA of β-catenin (si-β-catenin) or si-Scramble (si-SC). We found that after β-catenin interfering, the β-catenin (*P* < 0.05) and SOX9 (*P* < 0.001) protein levels were significantly downregulated (Additional file 2: Fig. S2C and D). These results suggested that miR-27b promotes chondrogenic differentiation and represses hypertrophy of hBMSCs, possibly through stimulating SOX9 expression via β-catenin pathway activation.

### *CBFB* is a novel target gene of miR-27b

**Fig. 6:**
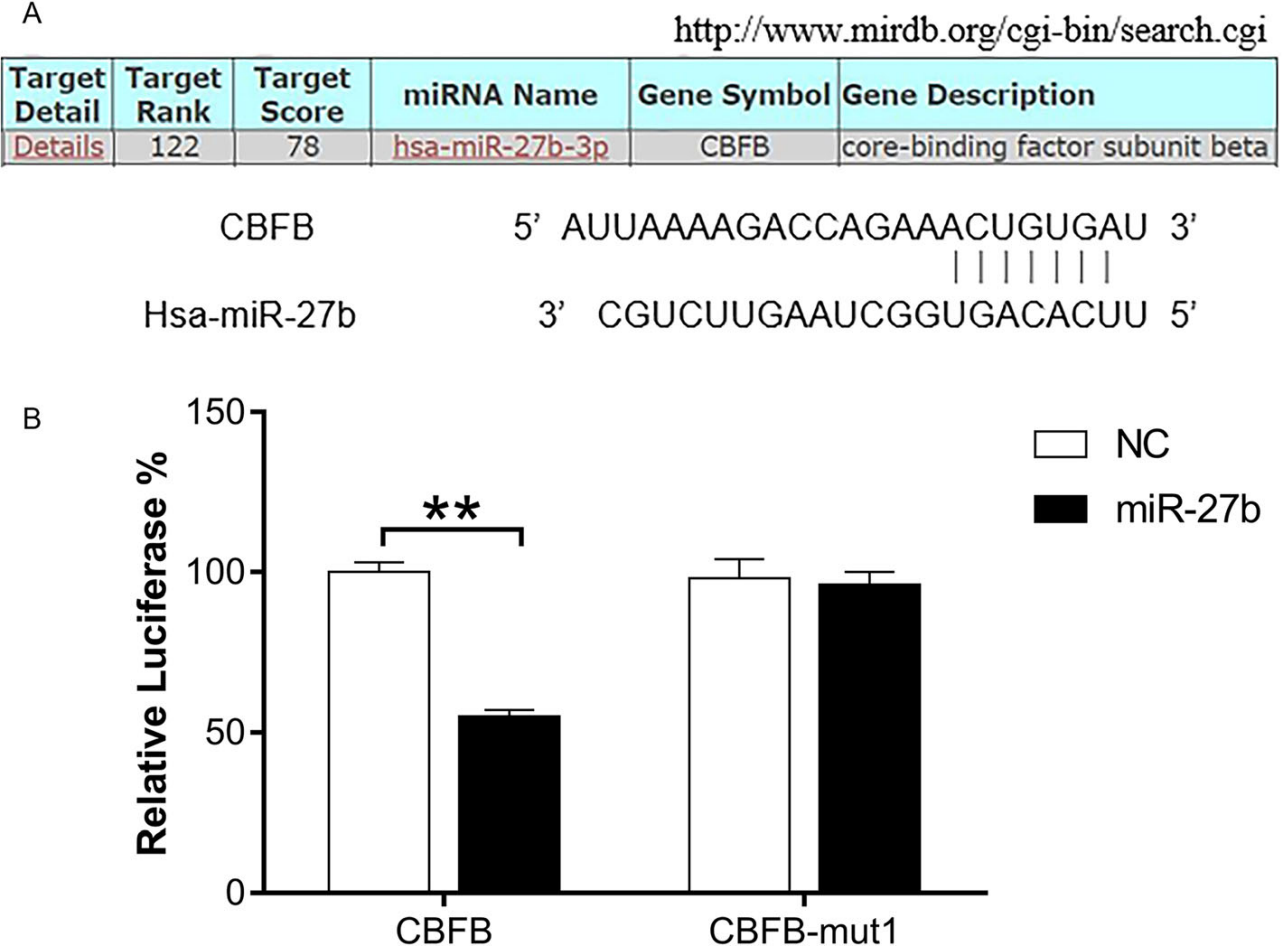
*CBFB* is a novel target gene of miR-27b. **a** Predicted binding sites in the 3′-UTRs of CBFB and miR-27b (microrna.org). **b** Luciferase activity of psiCHECK *CBFB* 3′-UTR. miR-27b distinctly suppressed the activity of the *CBFB* 3′-UTR, whereas *CBFB*-mut1 did not. **P < 0.001

Target prediction using two different tools [TargetScan (www.targetscan.org) and miRDB (www.microrna.org)] suggested that *CBFB* is a potential target of miR-27b (Additional file 3). In addition, it showed that the binding sites for miR-27b are evolutionarily conserved between humans and rats. CBFB is predicted to be targeted by miR-27b-3p in miRDB (Fig. 6a). Using RT-qPCR and western blotting, we found that overexpression of miR-27b resulted in significantly decreased mRNA and protein levels of CBFB (Fig. 4a–c). In a luciferase reporter assay, CBFB-luciferase activity was significantly reduced in the presence of pre-miR-27b mimic (*P* < 0.001), whereas there was no significant effect when *CBFB* carried a point mutation (Fig. 6b). This demonstrated that *CBFB* was a target of miR-27b, and miR-27b can directly bind to the 3′-UTR region of *CBFB* mRNA and downregulate its expression.

### Overexpression of miR-27b in hBMSCs inhibits their hypertrophic differentiation and enhances their cartilage defect repair efficacy in vivo

**Fig. 7:**
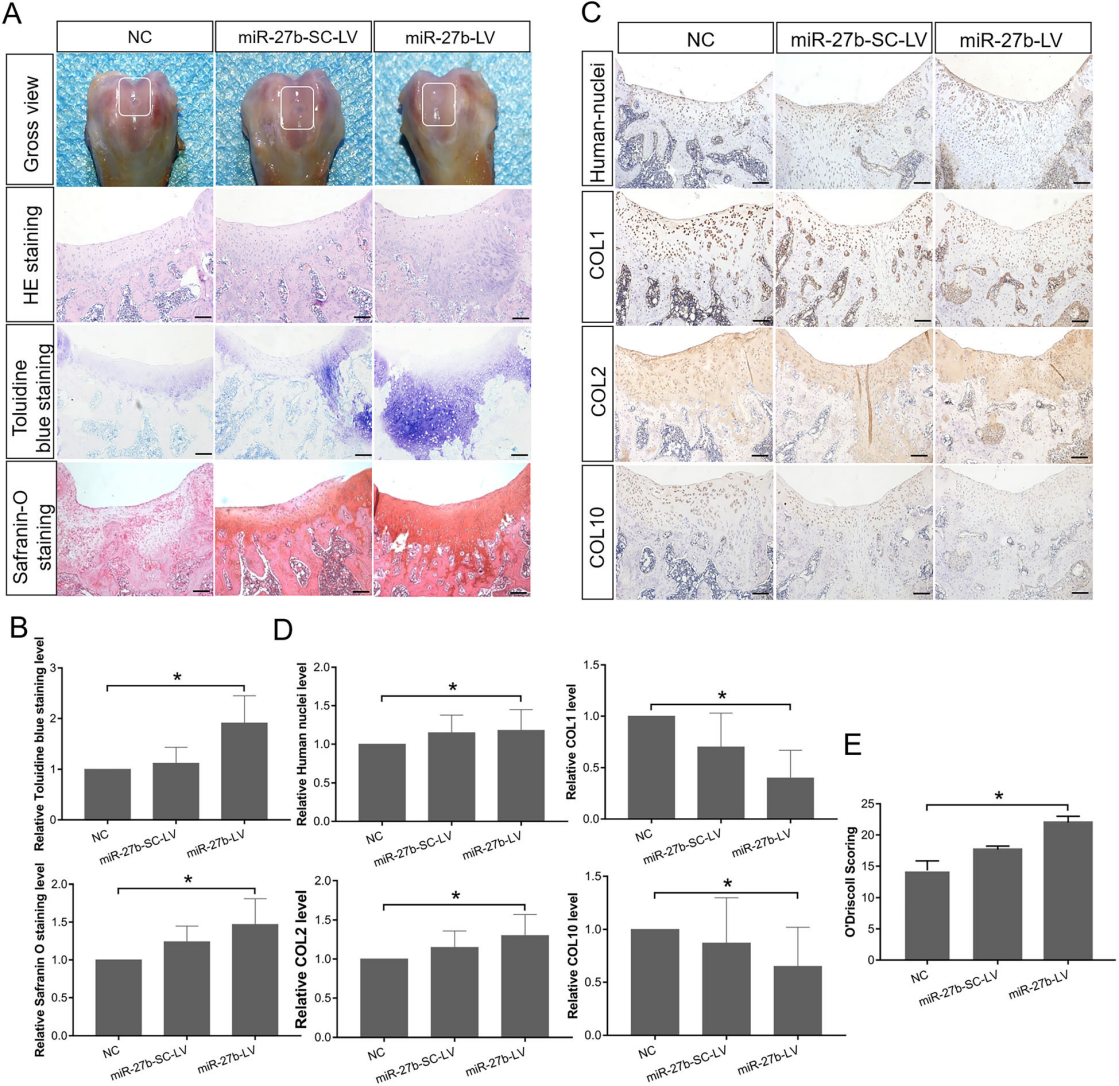
miR-27b overexpression in hBMSCs inhibits their hypertrophic differentiation and enhances their ability to repair cartilage defects in a rat model. **a** Gross photos of articular cartilage defects in a rat model. Cartilage pellets induced from hBMSCs transfected with NC, miR-27b-SC-LV, and miR-27b-LV group (*n* = 6 in each group) were transplanted into model rats with full-thickness articular cartilage lesions. HE, Toluidine blue, and Safranin O staining of femoral sections. Scale bar = 100 μm. **b** Safranin O staining and Toluidine blue staining were performed to measure levels of GAGs and proteoglycans. Optical density for GAGs and proteoglycans was evaluated by ImageJ software, and the data represented GAG and proteoglycan content. **c** IHC staining of human nuclei, COL1, COL2, and COL10 in femoral sections to evaluate repair efficacy. Scale bar = 100 μm. **d** Optical density for human nuclei, COL1, COL2, and COL10 was evaluated by ImageJ software, and the data represented the expression levels of human nuclei, COL1, COL2, and COL10. *P < 0.05, **P < 0.001. **e** Mean histological scores obtained using the O’Driscoll score for cartilage repair at 4 weeks. *P < 0.05

To investigate miR-27b inhibition of hBMSC hypertrophic differentiation in a cartilage defect environment in vivo, we established a full-thickness articular cartilage defect in rats and transplanted cartilage pellets induced from hBMSCs. Three experimental groups were included: a NC group, a miR-27b-SC-LV group, and a miR-27b-LV group. Femoral samples were subjected to histological analysis using Toluidine blue and Safranin O staining (Fig. 7a, b), as well as IHC (Fig. 7c, d). Histologically, in the miR-27b-LV implants, the neogenerated tissue had a smoother surface, showed better integration with the surrounding normal cartilage, and maintained a relatively intact cartilage structure, with well-differentiated chondrocytes surrounded by abundant matrix proteoglycan. In contrast, in the miR-27b-SC-LV and NC groups, we observed nearly complete cartilage destruction, including severe fibrillation, obvious proteoglycan loss, and fewer chondrocyte clusters. IHC revealed strong COL2 expression (*P* < 0.05), but low expression of COL1 (*P* < 0.05), a fibrous chondrocyte marker, in the miR-27b-LV group. In contrast, in the NC and miR-27b-SC-LV groups, COL1 (*P* < 0.05) and COL10 (*P* < 0.001) expression was higher than that in the miR-27b-LV group. Finally, human cells in the cartilage lesion site were detected using an anti-human-nuclei antibody, showing that human cells localized within the implant site and in neogenerated tissue. Using a well-established histological grading scale (O’Driscoll score) [36], we evaluated the degree of articular cartilage repair among the different animal groups. We found that the histological grading of the miR-27b-LV group was significantly higher than that of the NC group and slightly higher than that of the miR-27b-SC-LV group without significant difference (Fig. 7e). Furthermore, immunohistochemical staining for osteopontin (OPN) and osteocalcin (OCN) showed that within the area adjacent to the hypertrophic zone of cartilage and subchondral bone, compared with the miR-27b-SC-LV group, the miR-27b-LV group exhibited less number of hypertrophic chondrocytes and bone marrow cavity structures forming, and more weakly positive OPN and OCN reactions, suggesting that miR-27b certainly tend to alleviate hypertrophic differentiation of hBMSC-derived chondrocytes and endochondral bone formation in vivo (Additional file 4: Fig S4). Collectively, these results demonstrated that ectopic expression of miR-27b in hBMSCs effectively alleviated hypertrophic chondrocyte differentiation and had tendency to enhance the repair efficacy of the hBMSCs in a rat cartilage injury model.

## Discussion

In the present study, we investigated the expression profile of miR-27b and some of its biological functions during hBMSC chondrogenic differentiation. We made the following observations: (1) during hBMSC chondrogenic differentiation, miR-27b expression declines over time and is negatively related with hypertrophic differentiation, (2) CBFB is a novel direct target of miR-27b, (3) artificially elevating miR-27b levels in hBMSCs inhibits the phenotype in hBMSC chondrocytes, (4) within a cartilage defect environment in vivo, miR-27b-overexpressing hBMSCs differentiate into non-hypertrophic chondrocytes and have improved repair efficacy compared with control hBMSCs. This study provided insights into the mechanism of hBMSC hypertrophic differentiation and new avenues for effective cartilage defect repair through hBMSC transplantation.

Accumulating evidence has shown that a number of miRNAs, including miR-140, miR-143, miR-145, miR-483, miR-181a, miR-218, and miR-410, play critical roles in hBMSC chondrogenic differentiation [15, 23, 25, 40, 44, 45]. The miR-140, miR-143, miR-145, miR-181a, and miR-221 expression patterns during hBMSC chondrogenic differentiation in our conditions were in line with those reported in previous studies [15]. Other investigators have reported that miR-145 target genes are involved in chondrocyte differentiation, including *SOX9*, *SMAD4*, and *CBFB* [20, 46, 47], and miR-140 target genes are *SMAD1* and *HDAC4* [39, 44]. During BMSC chondrogenic differentiation, miR-145 downregulation over time gradually reduces the inhibition of translation of its target genes. *SOX9* is a master gene in the regulation of chondrogenesis and the maintenance of the chondrocyte phenotype. In contrast, *SMAD4* and *CBFB* are involved in hypertrophic differentiation of chondrocytes [48]. We found that at 14 days of pellet culture, despite maintaining high *SOX9* mRNA expression, *COL10*, *RUNX2*, and *CBFB* expression levels were significantly enhanced, indicating that the cells had already begun hypertrophic differentiation. Through targeting *SMAD1* and *HDAC4*, miR-140 inhibits hypertrophic differentiation [39, 44]. Strikingly, the miR-140 expression pattern seems to contradict this function. Combining our data with data from other groups, we speculate that during hypertrophic chondrocyte differentiation, the consistent upregulation of miR-140 expression, at least through inhibiting target gene translation, partially mitigates the progression to hypertrophy. Nevertheless, other mechanisms involving unidentified miRNAs or target genes downregulate protein expression and counteract this effect, allowing BMSC chondrogenesis to trend toward hypertrophy. Therefore, the miRNA expression pattern during cell differentiation provides important clues for further, more detailed and more functional investigation.

In the present study, we focused on miR-27b as a novel miRNA involved in BMSC hypertrophic chondrocyte differentiation. From 7 days post induction, the hypertrophic markers RUNX2 and COL10 were significantly upregulated. Furthermore, after artificially enhancing miR-27b expression in hBMSCs, the protein levels of both of SOX9 and COL2 were increased, whereas those of RUNX2 and COL10 level were decreased. Combined with the in vivo data, these results indicate that low-level expression of miR-27b in hBMSCs during chondrogenic differentiation may contribute to hypertrophic differentiation. Consistent with that hypothesis, a previous study showed that during chondrogenic differentiation of hBMSCs, miR-27b expression tends to be upregulated. While no previous study has compared miR-27b expression between hBMSC-derived hypertrophic and normal chondrocytes, miR-27b has been reported as a key regulatory factor in chondrocyte development and differentiation [49]. We previously reported that in rat knee hyaline cartilage, chondrocytes in the superficial zone of articular cartilage had higher expression of miR-27b and miR-27a than did those in the hypertrophic zone. Further, we observed that when miR-27b is overexpressed in hypertrophic chondrocytes, the hypertrophy marker COL10 protein level declined, and inversely, SOX9 and COL2 protein levels increased [26]. Consistent with those observations, in superficial zone cartilage of human limbs, miR-27 levels were approximately 2–3 times higher than in hypertrophic zone cartilage [50]. Based on these and our findings, we suggest that maintaining a high level of miR-27b in chondrocytes, including those derived from hBMSCs, is essential for inhibiting hypertrophic differentiation and maintaining the resting status of chondrocytes.

Like other miRNAs, miR-27b has multiple target genes, including *MMP13*, *PPARγ2*, *MEF2C*, *SP1*, *PHB*, and *PAX3* [26, 27, 49, 51–55]. The identification of target genes of miR-27b involved in hBMSC chondrocyte differentiation is significant for overcoming hypertrophic differentiation. In this study, we observed that during hBMSC chondrogenic differentiation, RUNX2 and COL10 showed similar gene expression patterns. Based on previous reports [14, 32], we hypothesized that if RUNX2 expression could be downregulated, hypertrophic differentiation would be attenuated. Consistent with those data, after miR-27b overexpression, the expression of RUNX2 protein significantly decreased. However, we found no putative miR-27b binding site in the 3′-UTR of RUNX2 mRNA. Therefore, we hypothesize that miR-27b indirectly represses RUNX2 protein expression. As is well known, CBFB cooperates with RUNX2 to form DNA-protein complexes and protects RUNX2 from degradation [29, 32]. Hence, we analyzed *CBFB* as a potential target gene of miR-27b. Based on target prediction, western blot, luciferase reporter, and co-IP analyses, we validated that *CBFB* is a direct target of miR-27b and indeed associates with RUNX2. Based on these results, we speculate that the increased level of miR-27b during differentiation effectively downregulates CBFB protein expression, leading to RUNX2 degradation and reduced RUNX2–DNA complex formation, resulting in the suppression of downstream gene expression. *MEF2C* [52] and *PPARγ* [56], which also play key roles in regulating hypertrophic chondrocyte differentiation, are both miR-27b targets. Although we did not determine the effect of miR-27b on MEF2C protein expression, we suggest that artificially enhanced miR-27b levels in hBMSCs may inhibit hypertrophic differentiation at least in part through simultaneously targeting *MEF2C*, *PPARγ*, and *CBFB* mRNAs.

SOX9 is a master transcription factor that is essential for chondrogenic differentiation and development from MSCs [57]. Its expression is high in mitotic and early prehypertrophic chondrocytes, but downregulated in hypertrophic chondrocytes [42, 58]. In this study, we confirmed that during the 21-day chondrogenic differentiation, SOX9 expression was significantly upregulated at day 7 and was maintained at a high level, with an expression pattern similar to that of *COL2*. These data suggest that SOX9 is required for the initiation and promotion of chondrogenic differentiation of hBMSCs. SOX9 directly regulates chondrocyte-specific proteins, including COL2, aggrecan, and related proteins [59]. Moreover, SOX9 and RUNX2 directly interact through their evolutionarily conserved high-mobility group and runt domains, and SOX9 represses RUNX2 activity [60]. We observed that upon increasing miR-27b expression in hBMSCs, *SOX9* and *COL2* levels were significantly upregulated, whereas *RUNX2* and *COL10* expression was suppressed, indicating that hypertrophic differentiation may additionally be blocked by SOX9 overactivation or suppression of RUNX2 action.

Multiple signaling pathways have been shown to affect SOX9 expression in chondrogenic differentiation and development, including Hedgehog, bone morphogenetic protein, FGF, Notch, and WNT [61, 62]. Additionally, SOX9 expression in chondrocytes is strictly controlled by other factors (e.g., HIF1α, miR-145, SOX5) [63–65]. Wnt signaling plays an important role during chondrogenesis and hypertrophic differentiation [43]. Multiple studies have shown that the genes encoding the Wnt signaling regulatory components GSK-3β, adenomatous polyposis coli, and Wnt3a are direct targets of miR-27b [66–68]. In addition, β-catenin expression is modulated by miR-27b [68], and in intestinal epithelium cells, β-catenin/TCF4 is required for SOX9 expression [69]. However, these studies on the relationship between miR-27b and Wnt signaling used cancer cell lines or normal cells other than hBMSCs. Here, we proved that miR-27b modulates SOX9 expression through regulating β-catenin expression during hBMSC chondrogenic differentiation. Given that SOX9 inhibits Wnt signaling by promoting β-catenin phosphorylation [70], there may exist a feedback loop between SOX9 and β-catenin. Future studies will need to investigate the detailed relationship between SOX9 and β-catenin after miR-27b treatment during hBMSC chondrogenic differentiation.

MicroRNAs have emerged as potent therapeutic targets in the treatment of multiple diseases and injuries. To effectively improve intercellular miRNA levels and enhance their therapeutic efficacy, multiple exogenous microRNA delivery systems have been developed, including lentiviral and adenoviral vectors [71], poly (lactide-co-glycolide) [72], and liposomes [73]. In this study, we selected lentiviruses as our vector for delivering exogenous miR-27b. Current lentiviral and adenoviral vectors all have limitations, including safety issues and loss of miRNA efficiency, which seriously impede the clinical application of miRNAs [74]. Therefore, safer and more efficient viral vectors have to be developed.

This study provides new insights into the mechanism of hBMSC chondrocyte differentiation that will aid in the development of novel strategies for the treatment of cartilage injury based on the transplantation of hBMSCs expressing high levels of miR-27b. However, it has some limitations. First, we only focused on a single miR-27b target gene, *CBFB*, to elucidate the mechanism through which miR-27b modulates hypertrophic differentiation. MicroRNAs have numerous target genes and modulate multiple signaling pathways. Therefore, the detection of ectopic miR-27b overexpression in hBMSCs through high-throughput mRNA and/or proteomic analyses will provide more detailed and comprehensive data for elucidating the functions and mechanisms of miR-27b. Second, we utilized the rat cartilage defect model, in which the injured cartilage environment completely varies from the pathology of OA; this difference may be a leading cause of hypertrophic chondrocyte differentiation in hBMSCs. Accordingly, another suitable animal OA model is required to transplant the hBMSCs overexpressing miR-27b into the OA cartilage defect site to investigate neocartilage properties and therapeutic efficiency. After conducting detailed mechanistic and preclinical animal studies, we can anticipate that miR-27b-expressing hBMSC transplantation will be an effective clinical therapy for cartilage injury, including OA.

## Conclusion

Maintaining high-level miR-27b expression in hBMSCs is necessary for inhibiting hypertrophic chondrogenesis and is a potential therapeutic option for articular cartilage repair and preventing OA defects.

## Supplementary information

**Additional file 1:** **Fig S1.** hBMSCs multidifferentiation potential. (A) Differentiation into osteoblasts as verified by alizarin red staining. After 4 weeks of induction, calcium nodules were observed. Scale bar = 100 μm. (B) Differentiation into adipocytes verified by Oil Red O staining. After 3 weeks of induction, intracellular lipid droplets were detected. Scale bar = 50 μm. (C) HE staining. Scale bar = 100 μm. (D) Alcian blue staining. Scale bar = 50 μm. (E) Safranin O staining. Scale bar = 50 μm.

**Additional file 2: Fig S2.** miR-27b unregulated SOX9 expression through β-catenin. (A) Protein levels of β-catenin and SOX9 in hBMSCs after being transfected with miR-27b mimic and scramble as measured by western blot. (B) Semi-quantification of western blot data. *P < 0.05, **P < 0.001. (C) Protein levels of β-catenin and SOX9 in hBMSCs transfected with miR-27b mimic, si-SC and si-β-catenin as measured by western blot. (D) Semi-quantification of western blot data. *P < 0.05, **P < 0.001.

**Additional file 3.** The prediction results of miR-27b binding site to 3`-UTR of CBFB mRNA.

**Additional file 4.** IHC staining of OPN and OCN for detecting endochondral bone formation in the hypertrophic cartilage region. Positive control is growth plate area in the same slide. Scale bar = 50 μm. The blank arrow denotes bone marrow cavity structures, and white arrow denotes the junction area between the hypertrophic zone of cartilage and subchondral bone. The black triangle denotes hypertrophic zone of cartilage in the positive control.

## Data Availability

All data generated or analyzed during this study are included in this published article.

## Abbreviations

BMSCs: Bone marrow mesenchymal stem cells
OA: Osteoarthritis
miR-27b: microRNA-27b
COL: Collagen
MMP13: Matrix metalloproteinase 13
IHH: Indian hedgehog
miRNAs: microRNAs
LncRNAs: Long noncoding RNAs
UTR: Untranslated region
HDCA4: Histone deacetylase 4
PPARγ: Peroxisome proliferator-activated receptor γ
CBFB: Core-binding factor, β-subunit
RUNX1–3: Runt-related transcription factors 1–3
FBS: Fetal bovine serum
bFGF: Basic fibroblast growth factor
EDTA: Ethylenediaminetetraacetic acid
PBS: Phosphate-buffered saline
TGF-β3: Transforming growth factor β3
NC: Negative control
co-IP: Coimmunoprecipitation
miR-27b-SC-LV: miR-27b-scramble-LV
HE: Hematoxylin and eosin
GAG: Glycosaminoglycan
IHC: Immunohistochemistry
chon: Chondrocytes
OPN: Osteopontin
OCN: Osteocalcin
