# Supplementary figures and images for: MicroRNA-27b targets CBFB to inhibit differentiation of human bone marrow mesenchymal stem cells into hypertrophic chondrocytes
Source: Stem Cell Res Ther. 2020 Sep 11;11:392. doi: 10.1186/s13287-020-01909-y (PMC7488425; doi:10.1186/s13287-020-01909-y)

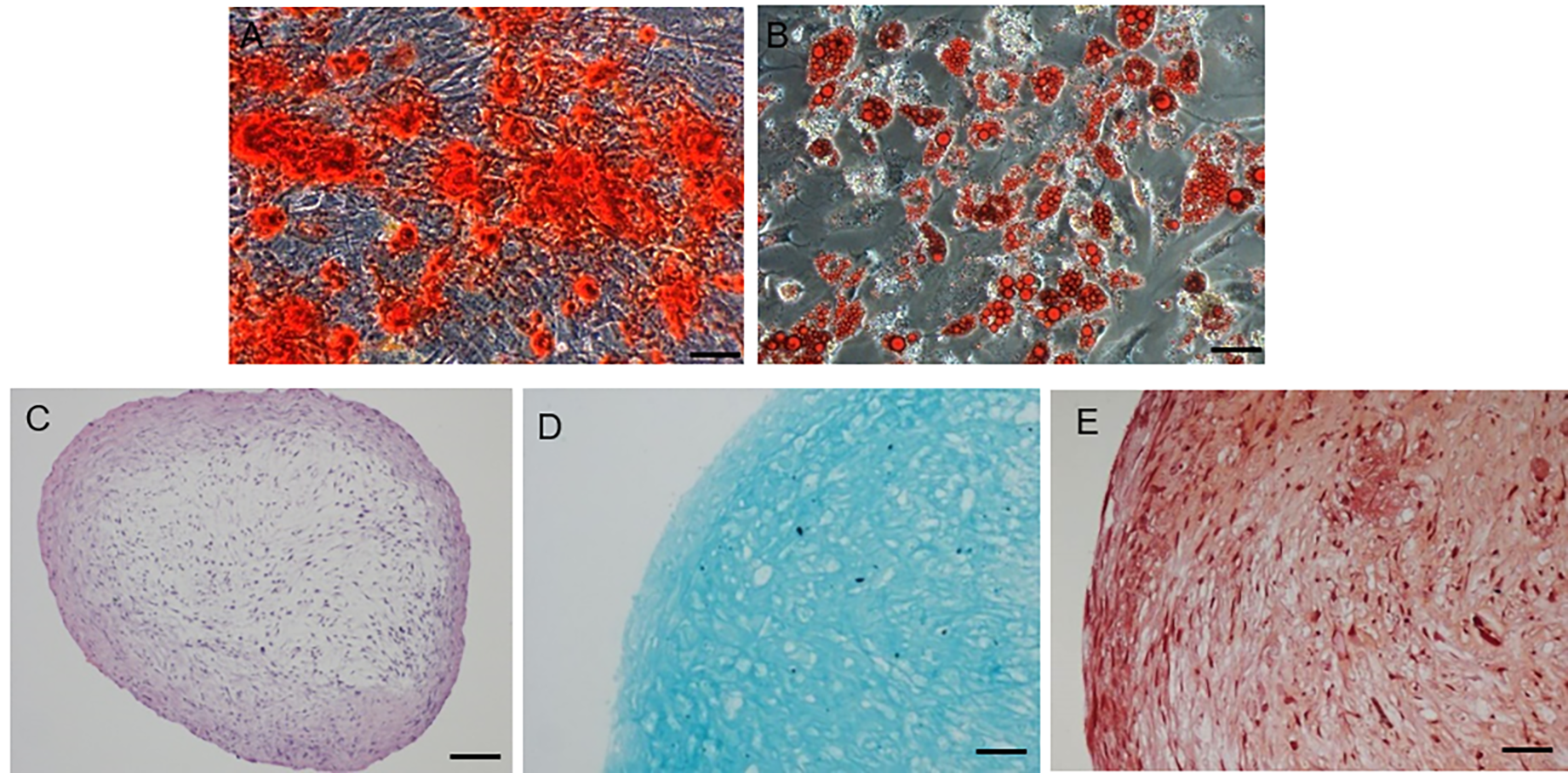

Supplement: Supplementary file 1 — Additional file 1: Fig S1. hBMSCs multidifferentiation potential. (A) Differentiation into osteoblasts as verified by alizarin red staining. After 4 weeks of induction, calcium nodules were observed. Scale bar = 100 μm. (B) Differentiation into adipocytes verified by Oil Red O staining. After 3 weeks of induction, intracellular lipid droplets were detected. Scale bar = 50 μm. (C) HE staining. Scale bar = 100 μm. (D) Alcian blue staining. Scale bar = 50 μm. (E) Safranin O staining. Scale bar = 50 μm. [file 13287_2020_1909_MOESM1_ESM.tif]

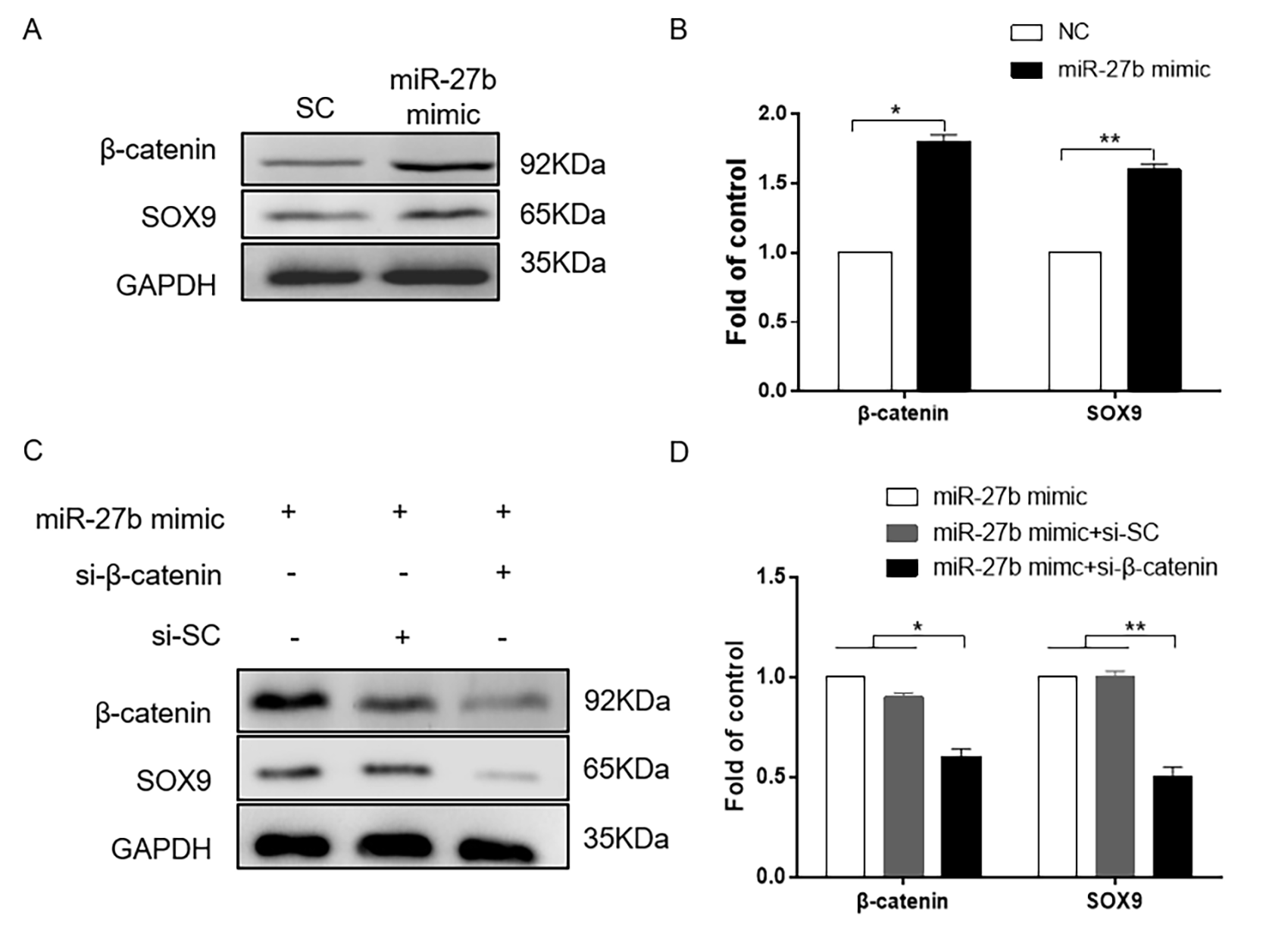

Supplement: Supplementary file 2 — Additional file 2: Fig S2. miR-27b unregulated SOX9 expression through β-catenin. (A) Protein levels of β-catenin and SOX9 in hBMSCs after being transfected with miR-27b mimic and scramble as measured by western blot. (B) Semi-quantification of western blot data. *P < 0.05, **P < 0.001. (C) Protein levels of β-catenin and SOX9 in hBMSCs transfected with miR-27b mimic, si-SC and si-β-catenin as measured by western blot. (D) Semi-quantification of western blot data. *P < 0.05, **P < 0.001. [file 13287_2020_1909_MOESM2_ESM.tif]

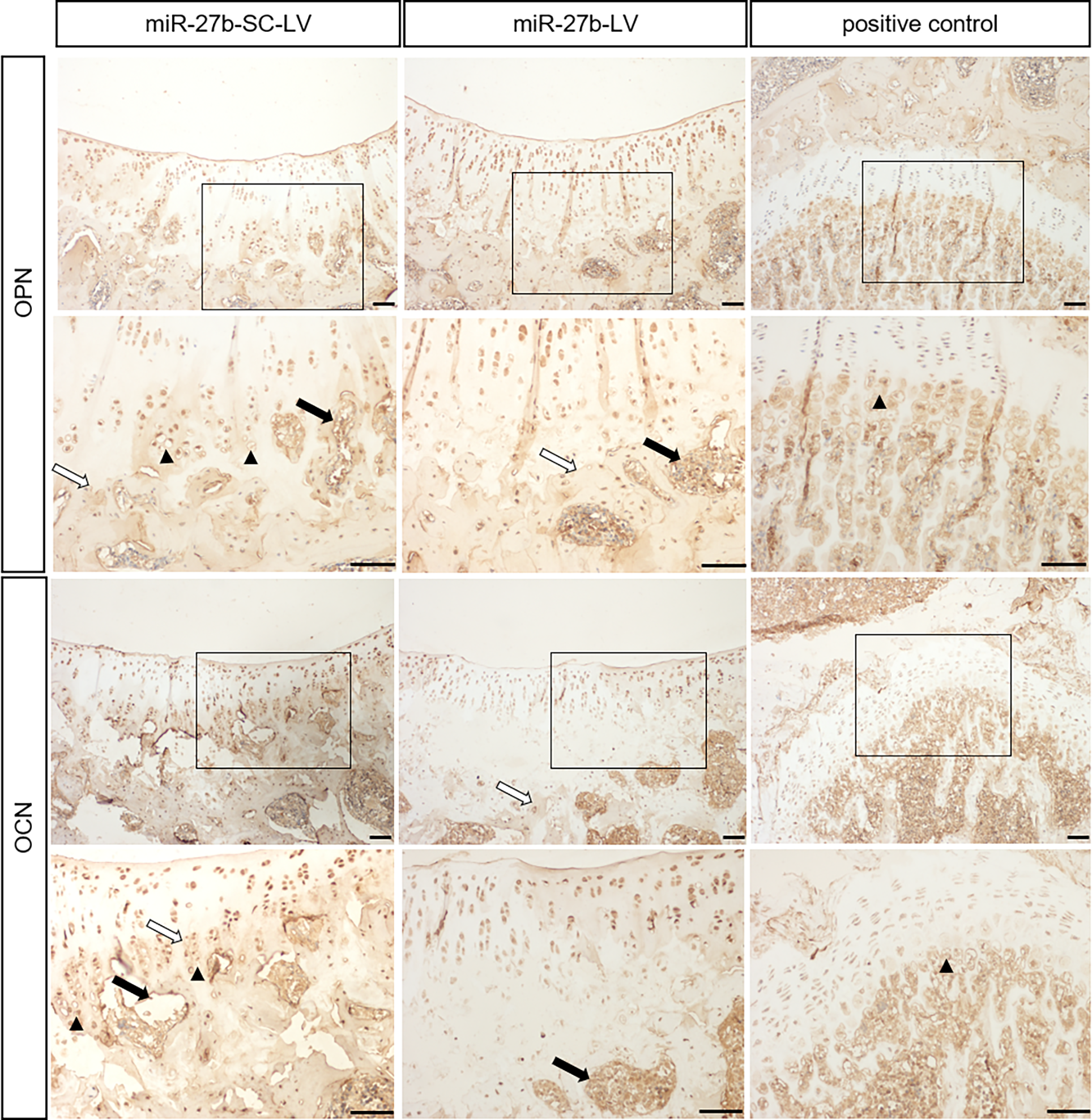

Supplement: Supplementary file 4 — Additional file 4. IHC staining of OPN and OCN for detecting endochondral bone formation in the hypertrophic cartilage region. Positive control is growth plate area in the same slide. Scale bar = 50 μm. The blank arrow denotes bone marrow cavity structures, and white arrow denotes the junction area between the hypertrophic zone of cartilage and subchondral bone. The black triangle denotes hypertrophic zone of cartilage in the positive control. [file 13287_2020_1909_MOESM4_ESM.tif]
