# Supplementary material for: MicroRNA-27b targets CBFB to inhibit differentiation of human bone marrow mesenchymal stem cells into hypertrophic chondrocytes
Source: Stem Cell Res Ther. 2020 Sep 11;11:392. doi: 10.1186/s13287-020-01909-y (PMC7488425; doi:10.1186/s13287-020-01909-y)

**The prediction results of miR-27b binding site to 3`-UTR of CBFB mRNA.**

1. TargetScan result (URL: http://www.targetscan.org/vert_72/):


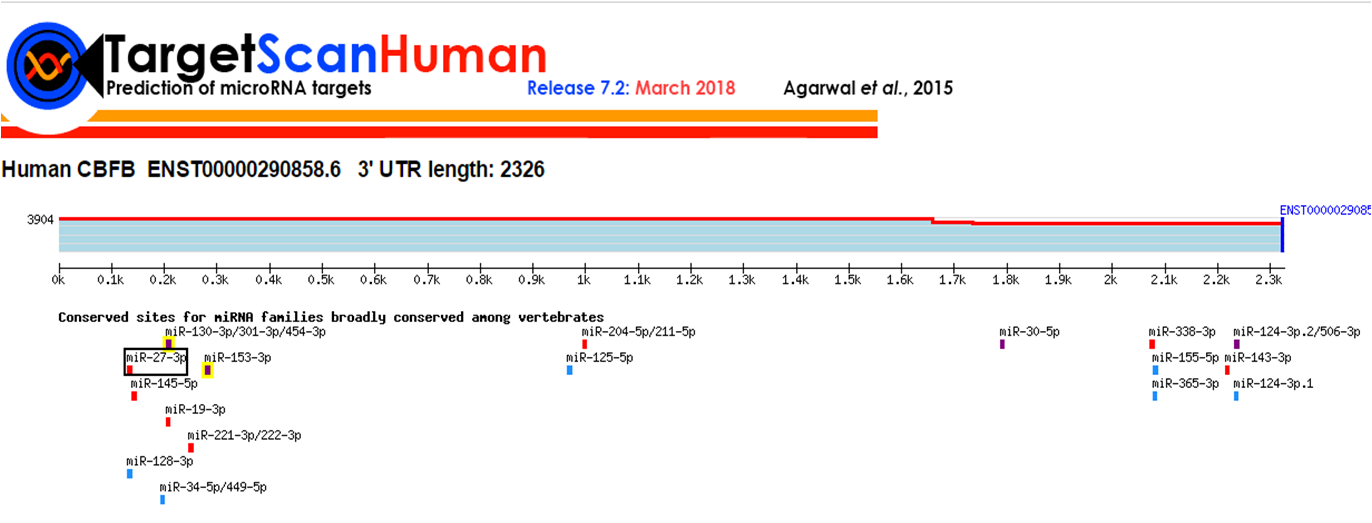


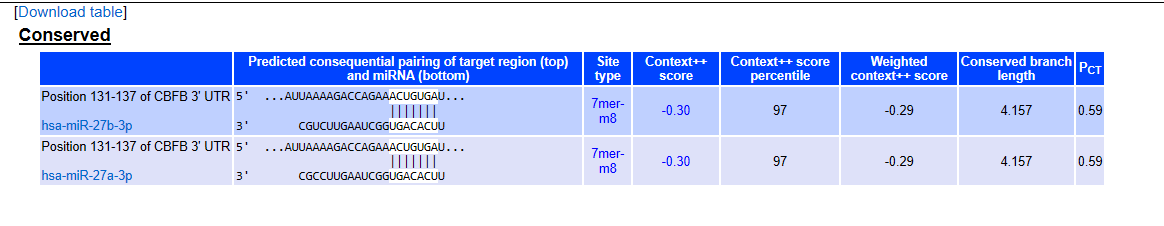


1. miRDB scanning result (http://mirdb.org/):


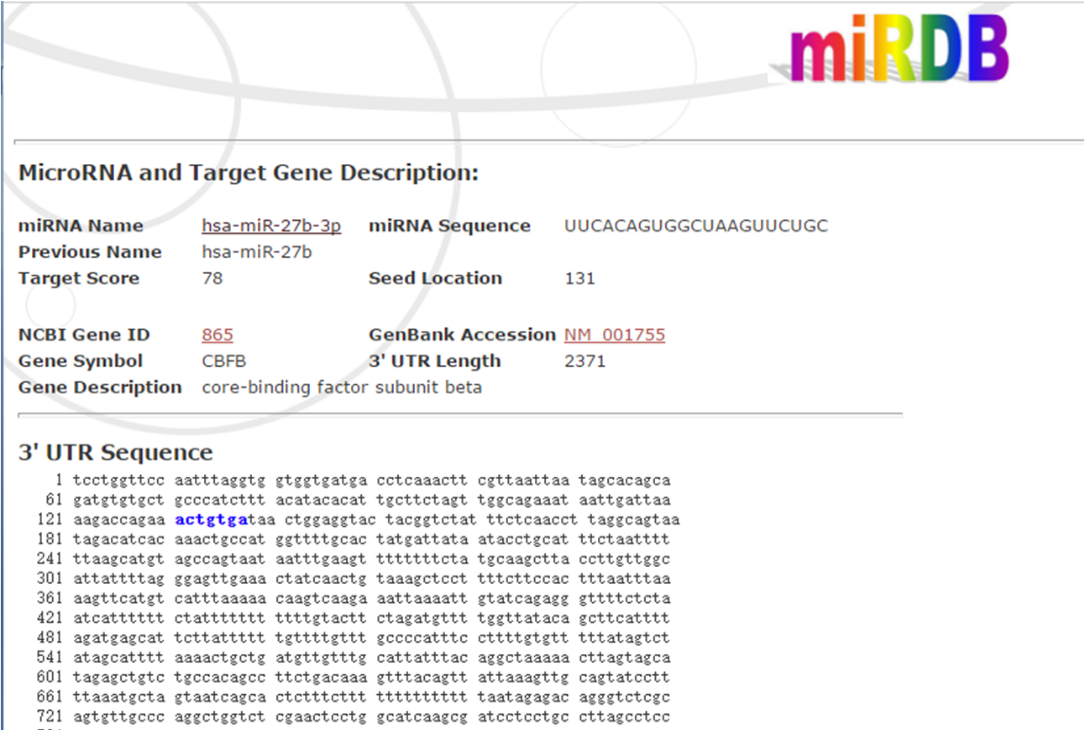


1. NCBI nucleotide BLAST result (https://blast.ncbi.nlm.nih.gov/Blast.cgi):


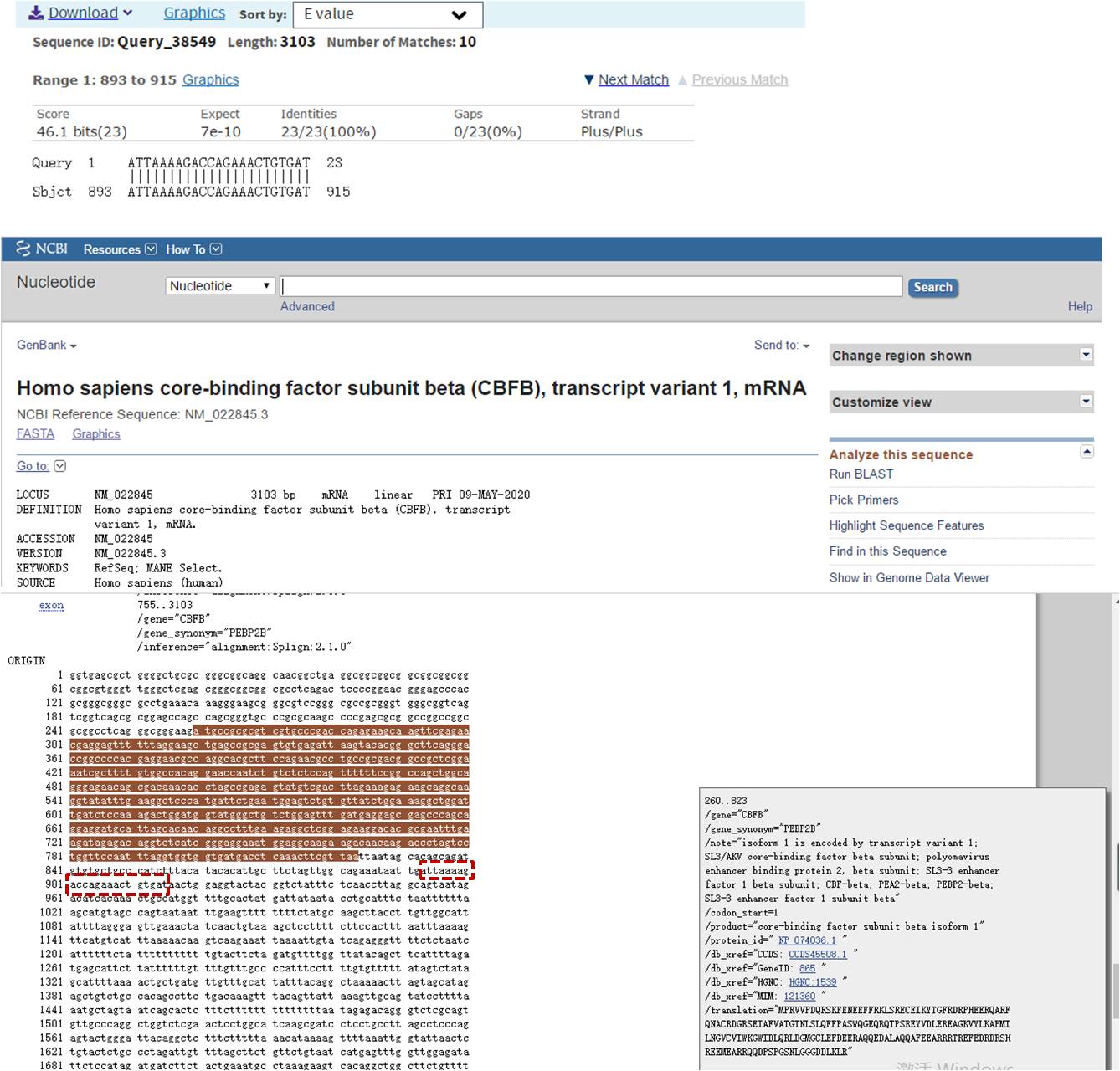

Supplement: Supplementary file 3 — Additional file 3. The prediction results of miR-27b binding site to 3`-UTR of CBFB mRNA. [file 13287_2020_1909_MOESM3_ESM.docx]
